# *Chlamydia trachomatis* Immune Evasion via Downregulation of MHC Class I Surface Expression Involves Direct and Indirect Mechanisms

**DOI:** 10.1155/2011/420905

**Published:** 2011-05-29

**Authors:** Joyce A. Ibana, Danny J. Schust, Jun Sugimoto, Takeshi Nagamatsu, Sheila J. Greene, Alison J. Quayle

**Affiliations:** ^1^Department of Microbiology, Immunology, and Parasitology, Louisiana State University Health Science Center, New Orleans, LA 70112, USA; ^2^Department of Obstetrics, Gynecology and Women's Health, University of Missouri School of Medicine, Columbia, MO 65202, USA

## Abstract

Genital *C. trachomatis* infections typically last for many months in women. This has been attributed to several strategies by which *C. trachomatis* evades immune detection, including well-described methods by which *C. trachomatis* decreases the cell surface expression of the antigen presenting molecules major histocompatibility complex (MHC) class I, MHC class II, and CD1d in infected genital epithelial cells. We have harnessed new methods that allow for separate evaluation of infected and uninfected cells within a mixed population of chlamydia-infected endocervical epithelial cells to demonstrate that MHC class I downregulation in the presence of *C. trachomatis* is mediated by direct and indirect (soluble) factors. Such indirect mechanisms may aid in priming surrounding cells for more rapid immune evasion upon pathogen entry and help promote unfettered spread of *C. trachomatis* genital infections.

## 1. Introduction

*Chlamydia trachomatis* is an obligate intracellular bacterium with a distinctive developmental cycle [1]. The bacteria alternate between two forms: the metabolically inactive, infectious elementary body (EB) and the metabolically active, noninfectious, intracellular reticulate body (RB). EBs infect permissive epithelial cells and differentiate into RBs within a host-derived vacuole which is termed an inclusion. After RBs multiply by binary fission they redifferentiate into EBs. Newly formed EBs are likely to disseminate *in vivo* via direct epithelial cell rupture and release of EBs into the extracellular milieu, or by extrusion of the intact inclusion or dislodgement of infected cells [2–4]. 

The worldwide burden of *C. trachomatis*-associated morbidity is great. *C. trachomatis* serovars A–C infect the conjunctival epithelium and cause trachoma, the major cause of preventable blindness worldwide [5]. *C. trachomatis* serovars D–F most commonly infect the columnar epithelium of the genital tract and chlamydial infection is the most prevalent bacterial sexually transmitted infection (STI) [6]. The endocervix is the primary point of entry, and most commonly infected site, in the female genital tract [6]. Genital infection in women is usually asymptomatic, but can lead to significant morbidity including ascending infection, pelvic inflammatory disease, chronic and debilitating pelvic pain, and tubal infertility [6]. The remarkable ability of *C. trachomatis* to infect the genital tract for extended periods of time, but characteristically cause asymptomatic infections, means that the pathogen goes undetected, and therefore untreated, in many women [6]. This increases the likelihood of secondary pathogen introduction, downstream pathological sequelae, and increased transmission to unsuspecting sexual partners. 

How *C. trachomatis *establishes these extended infections is not completely understood, but this has been attributed to several immune evasion strategies employed by the pathogen [6]. We and others have described downregulation of antigen presentation by *C. trachomatis*-infected epithelial cells as a means of evading immune detection by both the innate and adaptive arms of the immune response [7–11]. These investigations were undertaken in mixed cultures of infected and uninfected cells [7–11]. Since only a proportion of epithelial cells will be infected by EB during any *in vivo *exposure event, we hypothesized that efficient and effective immune evasion early in the infectious process might involve effects on antigen presentation both in the cell directly infected by the pathogen and in surrounding cells that might be infected in subsequent rounds of infection. Here, we used state-of-the-art methods to isolate and separately analyze *C. trachomatis*-infected endocervical epithelial cells and noninfected bystander endocervical cells that were exposed to infected cells in the same culture. We evaluated MHC class I surface expression in these two cell populations to determine whether *C. trachomatis*-associated downregulation of MHC class I occurs in noninfected, but exposed bystander cells. All experiments were undertaken in the A2EN cells, a human immortalized endocervical epithelial cell line we recently established that retains site-appropriate expression of key differentiation proteins and components of the innate epithelial cell response [12]. 

## 2. Materials and Methods

### 2.1. Cell Culture Conditions and *C. trachomatis* Infection

The A2EN human endocervical epithelial cell line was propagated in an antibiotic-free keratinocyte serum-free medium (KSFM, Invitrogen, Carlsbad, CA, USA) supplemented with 30 *μ*g/mL recombinant epidermal growth factor (rEGF; Invitrogen), 0.1 ng/mL bovine pituitary extract (Invitrogen) and 0.4 mM CaCl_2_ (Sigma, St. Louis, MO, USA) as previously described [12]. KSFM with the supplements is referred to here as complete KSFM (cKSFM). The cells were incubated to grow under humidified conditions with 2% O_2_ and 5% CO_2_ at 37°C.* C. trachomatis *infections were performed using serovar D (D/UW-3/Cx) in SPG (10 mM sodium phosphate (pH 7.2), 0.25 M sucrose, 5 mM L-glutamic acid) at an MOI of 1 to 3 as previously described [13]. In all experiments, a mock-infected control was included. Immediately after infection, SPG was removed and replaced with cKSFM alone or with cKSFM supplemented with 30 ng/mL interferon gamma (IFN*γ*; PeproTech, Rocky Hill, NJ, USA). Cells for immunofluorescent staining were cultured in 12-well culture plates with coverslips. Cells for flow cytometric analyses were cultured in 6-well culture plates and harvested using a mild cell detaching agent, Accutase (Innovative Cell Technologies, San Diego, CA, USA) at 38 hours after infection (hpi). 

### 2.2. Immunofluorescent Staining

Mock-infected and *C. trachomatis*-infected cells grown on coverslips were washed with phosphate buffered saline (PBS) and fixed with 4% paraformaldehyde. After fixation, the cells were washed and permeabilized with 0.5% saponin. Adherent cells were then blocked with Background Sniper blocking reagent (Biocare Medical, Concord, CA, USA) to inhibit nonspecific staining. To visualize the chlamydial inclusions, *C. trachomatis*-infected cells were stained using MeriFluor antichlamydial LPS conjugated to fluorescein isothiocyanate (Meridian Bioscience, Inc., Cincinnati, OH, USA). DAPI (Thermo Fisher Scientific, Rockford, IL, USA) was used to stain nucleic acid. Stained cells were fixed with Prolong Gold antifade reagent (Invitrogen). Images of the cells were acquired at 40x magnification using a fluorescent microscope (Olympus, Center Valley, PA, USA). 

### 2.3. Flow Cytometric Analyses of *C. trachomatis*-Infected and Uninfected Cells

Mock-infected A2EN cells and A2EN cells infected with *C. trachomatis* at MOIs of 1, 2, and 3 were harvested, fixed, and permeabilized using Perm/fix reagent (BD Biosciences, San Jose, CA, USA). The cells were then stained with anti-chlamydial-LPS-FITC (Accurate, Westbury, NY, USA) and analyzed by flow cytometry. Uninfected cells were delineated from* C. trachomatis-* infected cells in *C. trachomatis*-infected cultures using the FlowJo software gating tool (Tree Star, Inc., Ashland, OR, USA), by setting a threshold based on the baseline fluorescent intensity of unlabeled-mock-infected controls. The percentages of infected cells from *C. trachomatis* cultures infected at different MOIs were determined by setting the gating tool on the population of cells with fluorescence intensity above the established threshold. 

### 2.4. MHC Class I Expression Analysis Using Flow Cytometry

A2EN cells were mock-infected or infected with *C. trachomatis* at an MOI of 2 and grown in cKSFM and or cKSFM with 30 ng/mL IFN gamma for 38 hpi. Cells were then harvested using Accutase (eBioscience, San Diego, CA, USA), permeabilized with Perm/Fix reagent (BD Biosciences) and double-stained with anti-chlamydial-LPS-FITC (Accurate) and anti-MHC class-I-PE (W6/32, eBiosciences). Immunostained cells were analyzed by flow cytometry. We first separated uninfected cells from *C. trachomatis*-infected cells using a FlowJo software gating tool based on fluorescence measured on the FL1 channel (FITC). MHC class I surface expression levels on uninfected and *C. trachomatis*-infected cells were then determined based on fluorescence intensity analyzed using the FL2 channel (PE). Results were depicted using histograms. 

### 2.5. Effects of Cell Culture Supernatants on MHC Class I Expression

Culture media from mock-infected and *C. trachomatis*-infected (MOI = 2) cultures grown in the presence or absence of 30 ng/mL of IFN gamma were aspirated from cultures at 38 hpi. Harvested media were then centrifuged at 300 × g and supernatants were collected and filter-sterilized through 0.2 *μ*m filter units. Filtered supernatants were then diluted with fresh culture media at a ratio of 1 : 5 and added to fresh monolayers of A2EN cells. A separate culture in media alone was included as an additional negative control. Cells were incubated for 24 hours and harvested for analysis of MHC class I expression using flow cytometry using the methods described previously. 

## 3. Results and Discussion

*In vitro* models of *C. trachomatis* infection can be modified to cause first-round infection of nearly all of the exposed cells, but only when high *C. trachomatis* MOIs are used and cyclohexamide is added to the culture, which inhibits cell growth but also host protein synthesis. More common infection protocols, particularly those performed in the absence of cyclohexamide, result in a mixed population of infected and uninfected cells. While the latter more closely resembles *in vivo *infection [14], the mixed population of cells generated through *in vitro* protocols is typically evaluated in bulk. Recent advancements in flow cytometric analysis of permeabilized and intracellularly immunostained cells have allowed separate evaluation of infected and noninfected but exposed cells within a *C. trachomatis*-infected *in vitro* model [15–17]. We are not aware of any reports that have harnessed these methods to evaluate differences in the characteristics of the resultant cell populations, particularly those grown in the absence of cyclohexamide. 

A2EN cells are of endocervical origin and, although immortalized, they retain the characteristics of primary human endocervical epithelial cells through multiple passages [12, 18] and can be infected with *C. trachomatis*. Since the endocervix is the primary site of *C. trachomatis* entry in humans [6], A2EN cells represent a highly physiologic *in vitro* cell model for the study of heterosexual transmission of *C. trachomatis*. 

**Figure 1:**
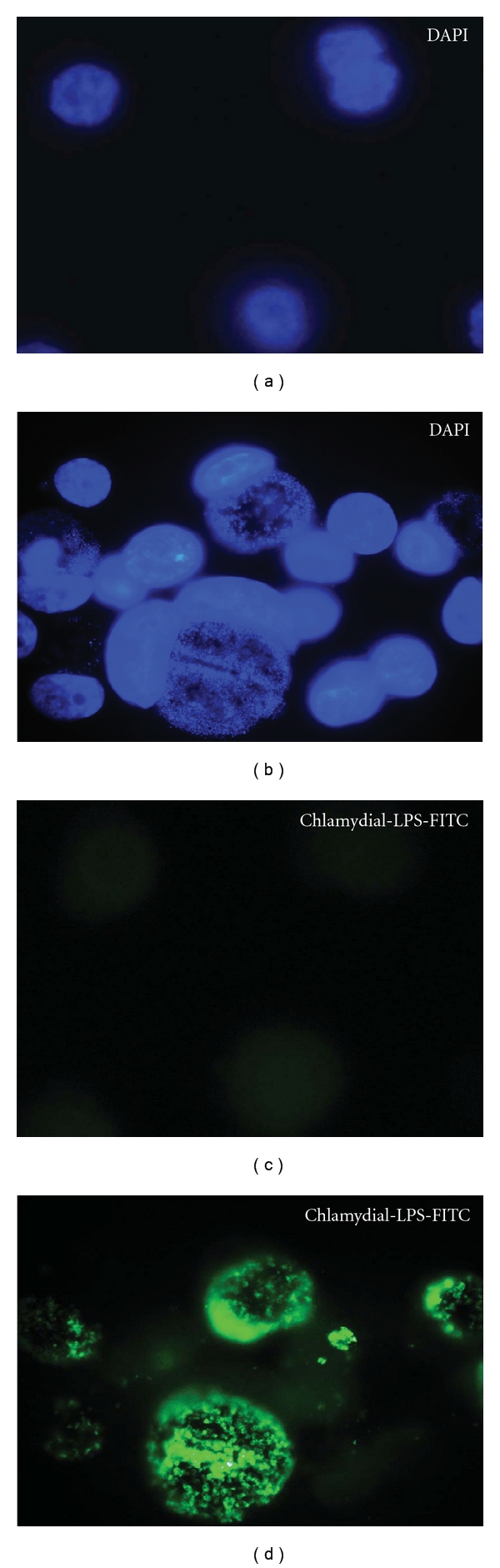
*C. trachomatis*-infected endocervical epithelial cell (A2En) culture. The immortalized endocervical epithelial cell line A2EN was exposed to *C. trachomatis* serovar D at multiplicity of infection of 2 in the absence of cyclohexamide (right two panels). This resulted in a mixed population of infected and uninfected cells. Infected cells are stained green with chlamydial LPS conjugated to FITC, and nucleic acid is stained with DAPI (blue). Uninfected controls are included in the left two panels for comparison. Data is representative of experiments performed greater than 10 times.

**Figure 2:**
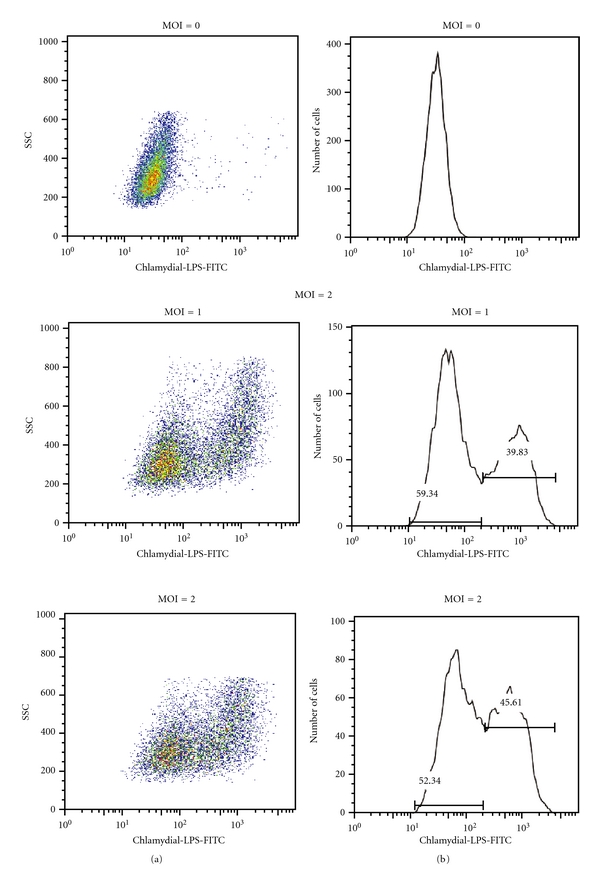

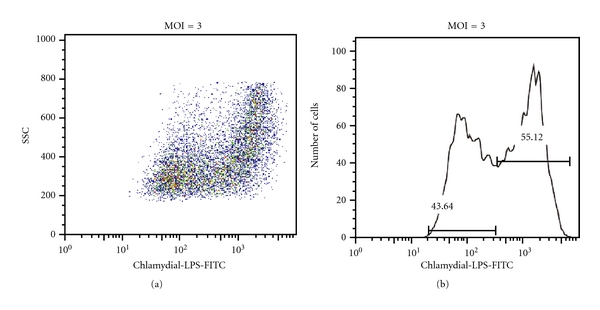
Separation of infected and uninfected endocervical epithelial cells in a *C. trachomatis-*exposed culture. Using chlamydial-LPS fluorescently labeled with FITC, infected and noninfected A2EN cell populations from the same culture can be distinguished and analyzed independently. This separation allows for the analysis of specific host cellular responses to *C. trachomatis *infection in infected and in bystander-uninfected cells. Left column shows side scatter (SSC) and chlamydial LPS-FITC dot plots, indicating a shift in granularity of FITC positive cells. Right column shows cell number and chlamydial-LPS histograms, which reflect the proportion of uninfected and C*. trachomatis*-infected cells within a pool of cells from A2En cultures exposed to varying multiplicities of infection (MOIs). Data is representative of experiments performed greater than 10 times.

We first verified flow cytometric separation of infected and exposed, noninfected A2EN cells using MOI titration experiments. A2EN cells were exposed to *C. trachomatis, *serovar D, one of the 3 most common genitally transmitted serovars in the United States [19] over a range of MOIs to generate mixed populations of infected and noninfected cells within a single culture. Cultures were then permeabilized, exposed to a FITC-labeled antibody against chlamydial lipopolysaccharide (anti-chlamydial-LPS-FITC) and evaluated using flow cytometry. Figure 1 depicts cells exposed to *C. trachomatis* serovar D at an MOI of 2 and cultured for 38 hpi. The upper panel shows a group of infected and uninfected A2EN cells stained with DAPI. The lower panel analyzes the same cells for FITC positivity and shows that *C. trachomatis* infection of A2EN cells at an MOI of 2 results in infection of approximately 40–50% of the cells in culture. Flow cytometric analysis of A2EN cells exposed to *C. trachomatis* serovar D at an MOI of 0 (control), 1, 2, or 3 and cultured for 38 hpi is shown in Figure 2. 

As in Figure 1, cells were permeabilized and immunostained with anti-chlamydial-LPS-FITC prior to flow cytometry. As expected, nonexposed cells showed no FITC-positive cells. As the *C. trachomatis* exposure MOI increases, the percentage of FITC-positive cells increases from 39.8% to 55.1%. The 45.6% LPS-FITC positivity noted after infection at an MOI of 2 confirms the nonquantitative results shown in Figure 1.

Methods that allow the distinct evaluation of *C. trachomatis*-infected versus uninfected but exposed cells within a single culture condition have many potential applications to human chlamydial disease. Our interest in chlamydial immune evasion of antigen presentation, combined with prior reports that infection of monocytes by *C. pneumonia* resulted in an IL 10-mediated downregulation of MHC class I expression in monocytes [20] led us to hypothesize that MHC class I downregulation in human endocervical cells may occur through direct and bystander mechanisms. 

**Figure 3:**
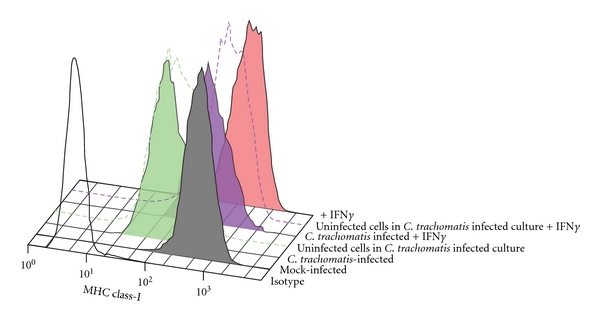
MHC class I surface expression on infected and uninfected bystander endocervical epithelial cells in cultures exposed to *C. trachomatis*. Surface expression levels of MHC class I were assessed in the two populations of cells found in *C. trachomatis*-exposed cultures. A2En cells were either mock infected (gray) or exposed to *C. trachomatis* at MOI of 2, with cKSFM alone (green) or cKSFM with 30 ng/mL IFN gamma (blue). Mock-infected A2EN cells exposed to 30 ng/mL IFN gamma (red) were analyzed in parallel. Isotype control is shown as black line histogram. Surface expression of MHC class I is depicted using histograms. The green solid histogram represents *C. trachomatis*-infected cells, the dashed green line represents bystander uninfected cells, the blue solid histogram represents *C. trachomatis*-infected cells exposed to IFN gamma, and the blue dashed line represents uninfected cells in IFN gamma-exposed cultures. Downregulation of MHC class I expression was observed on both the *C. trachomatis*-infected cells and on bystander cells. Data is representative of experiments performed in duplicate three times.

In Figure 3, A2EN cells were mock infected or infected with *C. trachomatis* serovar D at an MOI of 2 for 38 hpi in the presence or absence of IFN*γ*. Control cultures were exposed to IFN*γ* alone. Cells were then permeabilized, exposed to anti-chlamydial-LPS-FITC and to a fluorescently-labeled antibody recognizing properly folded MHC class I molecules in association with *β*2-microglobiulin, and analyzed by flow cytometry. *C. trachomatis*-infected cells within the mixed culture had decreased cell surface expression of MHC class I when compared to mock-infected cells. Those infected cells exposed to IFN*γ* displayed an intermediate level of MHC class I expression when compared to uninfected, IFN*γ*-exposed cells and to *C trachomatis*-infected, non-IFN*γ*-exposed cells. A noninfected culture was exposed to IFN*γ* as a positive control and displayed the highest level of cell surface-expressed MHC class I molecules. Uninfected cells within the infected cultures also displayed reduced levels of surface MHC class I expression when compared to cells exposed to IFN*γ* alone (positive control) and to mock-infected cells. In both IFN*γ*-exposed and nonexposed cultures, the decrease in MHC class I expression in infected cells was greater than that in noninfected cells in the same culture. 

**Figure 4:**
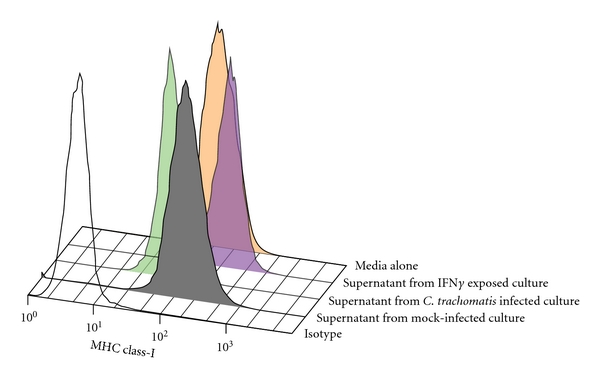
Effect of *C. trachomatis*-exposed A2EN cell culture media on MHC class I surface expression on uninfected A2EN endocervical epithelial cells. Uninfected monolayers of A2EN cells were exposed to culture media from *C. trachomatis*-infected A2EN cell culture (green), mock-infected culture (gray), IFN gamma exposed infected cell culture (blue), and fresh culture media (red). An isotype control is shown as black line histogram. Exposure of endocervical cells to culture media from *C. trachomatis*-infected culture at an MOI of 2 for 38 hours, (during which ~50% of cells are infected with *C. trachomatis)* resulted in the downregulation of MHC class I expression on endocervical epithelial cells. Data is representative of experiments performed twice in duplicate.

To confirm that these effects were the result of soluble mediators, uninfected A2EN cells were exposed to fresh culture media, to media from mock-infected cells (38 hpi) and to media from cells infected by *C. trachomatis* serovar D at an MOI of 2 for 38 hours in the presence and absence of IFN*γ*. Cells were then immunostained for properly folded MHC class I as above and analyzed by flow cytometry (Figure 4). 

A2EN cells exposed to supernatants from noninfected, IFN*γ*-exposed cells exhibited the greatest increase in MHC class I surface expression when compared to those exposed to media alone or to supernatants from mock-infected controls. Those exposed to supernatants from *C. trachomatis *serovar D-infected cells had a marked decrease in MHC class I surface expression, confirming that downregulation of MHC class I can be mediated by a purely soluble factor.

Zhong et al. initially described immune evasion through downregulation of antigen presentation in *C. trachomatis*-infected cells in 1999 [9]. They first reported decreased surface expression of interferon gamma-induced MHC class II molecules in human breast (MCF-7) and ectocervical (HeLa) cells infected with *C. trachomatis* serovar L2 [9] and subsequently described the downregulation of IFN gamma-induced and constitutive expression of MHC class I using HeLa cells and *C. trachomatis* serovar L2 [10]. They have further defined the mechanism of downregulation of both MHC class I and class II expression to involve a chlamydia-specific protease called chlamydial protease activity function (CPAF) [8]. We have shown that CPAF is also involved in the downregulation of surface expression of the MHC-like lipid antigen-presenting molecule, CD1d, in human penile urethral epithelial cells [7]. CD1d downregulation has functional implications in that *C. trachomatis* serovar F infection inhibits CD1d ligand-induced secretion of IL-12 and IL-15, but not of IL-10 [11]. It is not surprising that all three antigen presenting systems are affected by *C. trachomatis* infection, as CD4+ cells, CD8+ cells, and NKT cells have been implicated in *C. trachomatis* clearance and/or pathogenesis [6, 21–26].

Modulation of antigen presentation in neighboring uninfected cells may be advantageous to chlamydial survival *in vivo*. Inhibition of a nearby cell's ability to rapidly detect infection should widen the time frame for unfettered spread of the infection. Our methods, which evaluate infected and uninfected bystander cells at 38 hpi, allow for the effects of soluble mediators to occur, which for proinflammatory cytokines and chemokines in *C. trachomatis* infection are delayed until 20–24 hours after infection [27]. We are presently in the process of further defining the host soluble factor or alternative factors that may be responsible for the decrease in surface expression of MHC class I among noninfected cells in *C. trachomatis* serovar D-infected endocervical cell cultures. We suspect there may be several factors involved. Results on *C. pneumonia*-induced downregulation of MHC class I expression in monocytes makes IL-10 a possible candidate mediator [20], and our own experiments using penile urethral cells infected with *C. trachomatis* serovar F infection suggest that IL-10 signaling may be specifically protected during genital chlamydial infections [11]. Others have shown that CXCL12 stimulation of CXCR4 on HeLa cells results in MHC class I ubiquitination and targeting to endosomal degradation [28]. Since we have noted a stimulation of CXCR4 surface expression in A2EN cells infected with *C. trachomatis* serovar D (unpublished observations), this raises the possibility that CXCL12 might also be another candidate mediator involved in MHC class I downregulation in our *in vitro* endocervical epithelial cell model. Rasmussen et al. [27] have shown that infection of transformed cervical and colonic epithelial cell lines with *C. psittaci* and *C. trachomatis* increases the secretion of IL-8, GRO*α*, GM-CSF, and IL-6, but only after 20–24 hours after-infection and these mediators will be studied in conjunction with the IL-10, CXCL12, and others to better define the mechanisms involved in MHC class I modulation in bystander cells within cultures of primary-like endocervical epithelial cells infected with genital serovars of *C. trachomatis.*
